# Organoids and Bioengineered Intestinal Models: Potential Solutions to the *Cryptosporidium* Culturing Dilemma

**DOI:** 10.3390/microorganisms8050715

**Published:** 2020-05-11

**Authors:** Samantha Gunasekera, Alireza Zahedi, Mark O’Dea, Brendon King, Paul Monis, Benjamin Thierry, Jillian M. Carr, Una Ryan

**Affiliations:** 1 Vector and Waterborne Pathogens Research Group, College of Science, Health, Engineering and Education, Murdoch University, Murdoch 6150, Western Australia, Australia; A.ZahediAbdi@murdoch.edu.au; 2 Antimicrobial Resistance and Infectious Diseases Laboratory, College of Science, Health, Engineering and Education, Murdoch University, Murdoch 6150, Western Australia, Australia; m.o’dea@murdoch.edu.au; 3 South Australian Water Corporation, Adelaide 5000, South Australia, Australia; Brendon.King@sawater.com.au (B.K.); Paul.Monis@sawater.com.au (P.M.); 4 College of Medicine and Public Health, Flinders University, Adelaide 5042, South Australia, Australia; jill.carr@flinders.edu.au; 5 Future Industries Institute and ARC Centre of Excellence for Convergent Bio and Nano Science, University of South Australia, Adelaide 5095, South Australia, Australia; benjamin.thierry@unisa.edu.au

**Keywords:** *Cryptosporidium*, three-dimensional intestinal model, in vitro, organ-on-a-chip, organoid

## Abstract

*Cryptosporidium* is a major cause of severe diarrhea-related disease in children in developing countries, but currently no vaccine or effective treatment exists for those who are most at risk of serious illness. This is partly due to the lack of in vitro culturing methods that are able to support the entire *Cryptosporidium* life cycle, which has led to research in *Cryptosporidium* biology lagging behind other protozoan parasites. In vivo models such as gnotobiotic piglets are complex, and standard in vitro culturing methods in transformed cell lines, such as HCT-8 cells, have not been able to fully support fertilization occurring in vitro. Additionally, the *Cryptosporidium* life cycle has also been reported to occur in the absence of host cells. Recently developed bioengineered intestinal models, however, have shown more promising results and are able to reproduce a whole cycle of infectivity in one model system. This review evaluates the recent advances in *Cryptosporidium* culturing techniques and proposes future directions for research that may build upon these successes.

## 1. Introduction

Parasites of the *Cryptosporidium* genus are among the main pathogens causing severe diarrheal disease and death in young children in developing countries [1]. In otherwise healthy individuals, *Cryptosporidium* infection is typically mild and self-limiting, however, in immunocompromised, malnourished, or very young patients the infection can be severe or even fatal [2]. The majority of *Cryptosporidium*-related deaths occur in children under five years of age, accounting for approximately 48,000 deaths in 2016 internationally within this age bracket. In Australasia, high-income North America, and high-income Asia-Pacific, there were no *Cryptosporidium*-related deaths in children under five years of age reported in 2016, whereas in the same year and age bracket, approximately 42,000 *Cryptosporidium*-related deaths occurred in sub-Saharan Africa. This highlights the reality that low-income countries bear a disproportionate burden globally for cryptosporidiosis, and sub-Saharan Africa is by far the most severely affected. In children, the disease burden of cryptosporidiosis is not limited to the span of acute diarrheal illness, with even mild or asymptomatic infection being associated with long-term consequences such as persistent nutrient malabsorption and stunted growth, which can cause significant harm later in life [3].

In developed nations, the burden of human cryptosporidiosis is highest in patients with human immunodeficiency virus and acquired immunodeficiency disease, although this is improving as a result of highly active antiretroviral therapy [4]. Most outbreaks occur due to contamination of recreational swimming pools or contact with either infected cattle or infected children [2]. The largest *Cryptosporidium* outbreak reported occurred as a result of contamination of the public water supply in Milwaukee, Wisconsin in 1993 and was estimated to have infected 403,000 people, causing 54 deaths and costing $96.2 million in medical costs and lost productivity [5,6,7]. Despite the global attention *Cryptosporidium* received as a result of this outbreak, cryptosporidiosis is still poorly controlled and continues to be under-diagnosed, with surveillance studies likely underestimating the true magnitude of outbreaks occurring in both developed and developing countries [2,8].

Of significance to global disease burden, there is currently no available vaccine for *Cryptosporidium* infection and very limited treatment options [9]. The drug nitazoxanide is the only pharmaceutical intervention approved by the US Food and Drug Administration to treat *Cryptosporidium* infection. However, it is only efficacious in immunocompetent patients, leaving those in most urgent need with no effective treatment [10]. In the absence of a vaccine or effective therapeutic, the best prevention methods against cryptosporidiosis are public health approaches such as basic personal hygiene, sanitation, and filtration or disinfection of the drinking water supply [2], highlighting a gap in the research sector in developing preventions and treatments.

Most *Cryptosporidium* infections in humans are caused by two species: *Cryptosporidium hominis* and *Cryptosporidium parvum* [11]. In addition to the inability to genetically manipulate *Cryptosporidium* until recently, and the inaccessibility of good animal infection models (particularly for *C. hominis*), the lack of a reliable method of in vitro propagation of either of these species has been a major reason why pharmaceutical and vaccine development has lagged behind other enteric pathogens of similar clinical importance [8,12]. Out of the four pathogens identified as causing the majority of moderate-to-severe diarrhea in children under five years of age (rotavirus, *Cryptosporidium*, *Shigella*, and enterotoxigenic *Escherichia coli* producing heat stable toxin) [1], *Cryptosporidium* is the only pathogen without either an effective vaccine or treatment [13,14,15].

### 1.1. Cryptosporidiosis

Transmission of *Cryptosporidium* mainly occurs via the fecal–oral route, most frequently through contact with an infected person or animal or as a result of ingesting contaminated food or water. There are subtle differences in the clinical presentation between *C. hominis* and *C. parvum* infection, with *C. hominis* being associated with more severe disease including non-gastrointestinal symptoms such as headache, fatigue, and joint pain, whereas *C. parvum* infection generally presents clinically as diarrhea only. Additionally, given that *C. hominis* and *C. parvum* have different host ranges, the risk factors for infection and modes of transmission are species-specific to an extent. The main reservoirs for *C. parvum* are animal species, while for *C. hominis* the most important reservoir is likely to be young children with asymptomatic infection [2].

The pathogenesis of cryptosporidiosis begins with parasite attachment and invasion of host enterocytes in the small intestinal lumen, which results in disruption of epithelial permeability, ablation of the brush border, and blunting of villi. Increased enterocyte turnover to replace damaged cells results in crypt hyperplasia. The loss of microvilli causes a reduction in the absorptive surface area and therefore reduces fluid and nutrient uptake which presents clinically as a high volume, watery diarrhea [16].

While cryptosporidiosis is predominantly a gastrointestinal disease, *Cryptosporidium* is able to complete its life cycle within the respiratory tract, causing respiratory cryptosporidiosis, which occurs mainly in immunocompromised patients [17,18,19]. Possible routes of infection include the spread of *Cryptosporidium* from the gastrointestinal tract to the respiratory tract through either circulation or inhalation of gastrointestinal contents during emesis. Person-to-person transmission may also occur through coughing. Respiratory cryptosporidiosis is uncommon in humans and represents a minor contribution to the epidemiology of disease caused by *Cryptosporidium* [20].

### 1.2. Life Cycle

**Figure 1:**
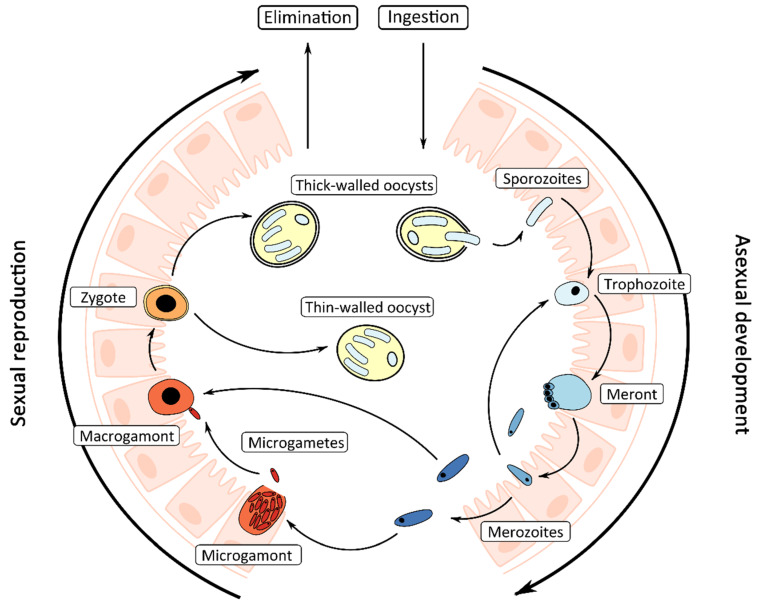
Diagrammatic representation of the *Cryptosporidium* life cycle in the intestine.

*Cryptosporidium* has a complex life cycle comprising both asexual (merogony) and sexual (gametogony) phases (Figure 1). The sporulated oocyst is the infectious environmental stage of the *Cryptosporidium* life cycle that is ingested by the host. Oocysts are highly resistant to standard disinfection practices and can survive for over a week in recreational water even at chlorine concentrations (>1–3 ppm) recommended by Centers for Disease Control and Prevention for maintenance of recreational swimming pools [2].

Excystation of the oocyst occurs in the intestine and is triggered by the temperature and pH conditions of the host’s gastrointestinal tract, resulting in the release of four motile sporozoites [21]. Sporozoites invade the host’s intestinal epithelial cells on the luminal surface, where they reside inside a parasitophorous vacuole which assumes an intracellular but extracytoplasmic position in the host cell. Formation of an attachment or feeder organelle is followed by the remainder of the asexual cycle, which includes development of trophozoites, meronts, and merozoites. Trophozoites undergo asexual proliferation by merogony to form meronts. Meronts develop four or eight nuclei, each incorporated into a merozoite, which are released from the parasitophorous vacuole once mature and later invade the surrounding host epithelial cells. These merozoites will either recycle as meronts and merozoites, or initiate the sexual cycle. During the latter, merozoites invade new host cells and either enlarge and develop into a uni-nucleate macrogamont or undergo cellular fission forming a multi-nucleated microgamont containing up to 16 non-flagellated microgametes. Microgametes are released from ruptured microgamonts; they penetrate host cells containing macrogamonts and subsequently fertilize the macrogamont forming a zygote. Productive fertilization fails to occur in vitro which is the main reason why in vitro propagation is inherently difficult [22]. In the host, the zygote undergoes meiosis, during which both thin-walled and thick-walled oocysts are formed, each containing four potentially infective sporozoites. Thin-walled oocysts remain within the host leading to autoinfection and persistent infections, and thick-walled oocysts are shed in the feces [23].

In a study by Hijjawi et al. [24], the presence of previously undescribed *Cryptosporidium andersoni* and *C. parvum* novel gamont-like extracellular stages were observed in in vitro cell culture. *C. andersoni* extracellular stages were also observed in cattle feces and were isolated using laser microdissection and subsequently sequenced and confirmed as *C. andersoni* [24]. These extracellular stages were described as gregarine-like due to their similarities to stages found in the life cycle of gregarines [25,26] and have been subsequently reported by Rosales et al. [27]. Despite being found in *C. andersoni* and *C. parvum*, which represent two very distinct lineages and most likely capture the range of phylogenetic diversity within the *Cryptosporidium* genus, the origin, significance, and fate of these stages in the *Cryptosporidium* life cycle and presence in broader *Cryptosporidium* spp. are unclear. Additionally, the current understanding of *Cryptosporidium* biology is incomplete, as *Cryptosporidium* was considered an obligate intracellular parasite until extracellular life cycle stages were observed and the ability for *Cryptosporidium* to complete its life cycle without host cells was reported [24,27,28]. Thus, there is detailed knowledge of the *Cryptosporidium* life cycle, but clear gaps in the basic understanding of this complex process.

### 1.3. In Vitro and In Vivo Models of Cryptosporidium Infection

In vivo models of *Cryptosporidium* infection have included neonatal calves, gnotobiotic piglets, and both immunodeficient and immunocompetent mice [29,30,31]. Neonatal calves experimentally infected with *C. parvum* are an effective animal model for human cryptosporidiosis, with the pathophysiology observed in calves being very similar to what is identified in humans [29]. Gnotobiotic piglets have been used to model infection with both *C. hominis* and *C. parvum* and, like humans, they are equally susceptible to both species [30]. However, their use is highly labor intensive and expensive, which limits the number of animals that are able to be used per experiment [30]. Immunocompetent mice are not susceptible to *C. hominis* or *C. parvum* infection, although neonatal and immunodeficient mice can be infected with *C. parvum*. Immunocompetent mice have been used to model infection with the natural mouse pathogen *Cryptosporidium muris*. However, *C. muris* is genetically distinct from both *C. parvum* and *C. hominis* and the gastric site of infection is different to the intestinal site of infection observed in human cryptosporidiosis [31]. A recent study was able to model *Cryptosporidium* infection using immunocompetent mice naturally infected with *Cryptosporidium tyzzeri* with both host and parasite amenable to genetic manipulation using a CRISPR/Cas9 based approach [32]. Using *C. tyzzeri* had several advantages over using *C. muris*, including the former’s genetic similarity to *C. parvum* and *C. hominis* and ability to cause similar pathology in mice to what is observed in cryptosporidiosis in humans. The use of reporter parasites expressing red fluorescent proteins mCherry and red-shifted luciferase has enabled precise quantification of parasite load as well as the ability to identify the location of infection in the immunocompetent mouse host accurately over time. The key host immune responses involved in parasite clearance were determined using a knockout-based approach in the mouse, which were identified to be interferon gamma (IFN-γ) and T cells [32]. Given that all models rely on immunocompromised or neonatal animals or animals that are naturally resistant to *C. parvum* and *C. hominis* infection, little is known about the adaptive immune responses required for lasting protection against *Cryptosporidium* infection.

In vitro models of *Cryptosporidium* infection have included the use of transformed cell lines which have been reviewed in detail elsewhere [33,34]. Long-term culture (over 25 days) has been reported using the human ileocecal adenocarcinoma (HCT-8) cell line after pH modification, gamma irradiation of the cell monolayer, and sub-culturing. However, fertilization is still not observed consistently and therefore this model does not reflect the complete *Cryptosporidium* life cycle [35]. More recently, the human esophageal squamous cell carcinoma (COLO-680N) cell line has been reported to support *C. parvum* infection and remain viable for eight weeks and was able to produce an oocyst yield of 1.2 × 10^7^ oocysts after 60 days, indicating that this cell line may be a promising option for future research [36]. The discovery that *Cryptosporidium* can complete its life cycle outside of a cellular host further justifies the use of an in vitro model over in vivo models [28], on top of the relative ease of use and lower cost.

## 2. Cell-Free Culture of *Cryptosporidium*

Hijjawi et al. [28] were the first to report that *C. parvum* is able to complete its life cycle in vitro in the absence of host cells. This was the first major breakthrough in culturing *Cryptosporidium* since 1984 when successful in vitro culture of *Cryptosporidium* was originally reported [37]. Axenic culture (consisting of just one species and entirely free of other contaminating organisms) of *C. parvum* was achieved by using a dual phase culturing system with a semi-solid layer of coagulated newborn calf serum underneath a suspension of purified oocysts in maintenance medium. Using this culturing method, fertilization was observed in vitro for the first time, and evidence of continuation of the *Cryptosporidium* life cycle was observed. Additionally, oocysts collected after 46 days of cell-free culture were infective to ARC/Swiss mice, demonstrating the in vitro production of infectious oocysts. Prior to that study, *Cryptosporidium* was considered an obligate intracellular parasite, thus the discovery that *Cryptosporidium* was able to complete its life cycle in the absence of host cells caused a paradigm shift in the understanding of the *Cryptosporidium* life cycle [28]. However, the findings of Hijjawi et al. [28] were initially controversial [38,39], with independent research groups either failing to establish *Cryptosporidium* growth in cell-free culture altogether or only being able to sustain growth for a short period of time [40,41]. In response to disputes of the legitimacy of the findings of Hijjawi et al. [28], an independent study used a combination of quantitative PCR and immunofluorescence to confirm that parasite multiplication was occurring when *C. parvum* was cultured in the absence of host cells [42]. These results were then validated by Hijjawi et al. [43], who additionally confirmed observations of *Cryptosporidium* life cycle stages using *Cryptosporidium*-specific antibody stains and fluorescent in situ hybridization probes.

Due to difficulty visualizing *Cryptosporidium* life cycle stages in cell-free culture compared to when grown in the presence of host cells, a study evaluating different staining methods found that life cycle stages were more readily able to be visualized with the use of antibody stains specific to *Cryptosporidium* such as Sporo-Glo™, known to bind to sporozoites, trophozoites, meronts, and merozoites [44]. Cell-free culture has also been validated using scanning electron microscopy and gene expression studies, which showed that gene expression patterns in *Cryptosporidium* were similar when cultured with and without host cells, but occurred at a slower rate with the latter [45]. Successful axenic culture of *Cryptosporidium* has since been reported by independent research groups [46,47,48,49,50] and life cycle stages have also been identified in a host cell-free biofilm environment [51]. In the study by Aldeyarbi & Karanis [48], evidence of the sexual stages of *C. parvum* development and the production of both thin- and thick-walled oocysts in cell-free culture were reported using transmission electron microscopy. Comparison of *Cryptosporidium* growth, both with and without host cells, has suggested that the host cells may enhance *Cryptosporidium* growth in vitro through the proteins they secrete into the growth medium and not necessarily through their role as a site of infection, though this finding requires additional confirmation [49,50]. Cell-free culture of *Cryptosporidium* has also enabled other aspects of parasite biology to be studied, including morphological changes that occur in sporozoites after excystation [52], the discovery of small molecules that induce parasite transformation from sporozoite to trophozoite [49], and several ultra-structural similarities to the Gregarines to be visualized [47]. The discovery that *Cryptosporidium* can be cultured without host cells has made an important contribution to the study of *Cryptosporidium* biology, though it remains controversial. To aid the study of host-parasite interactions and the pathophysiology of cryptosporidiosis, alternative culturing methods have gained momentum in recent years.

## 3. Bioengineered Intestinal Models for Culturing *Cryptosporidium*

Over the last five years, most attempts to grow *C. parvum* throughout its life cycle and produce new oocysts in vitro have centered around the use of bioengineered intestinal models involving perfusion or co-culture of various cells [53,54,55], including both mouse and human-derived intestinal organoids [56,57]. The pioneering work using a three-dimensional intestinal model to investigate *Cryptosporidium* infection utilized HCT-8 cells cultured in a low-shear, microgravity environment. Under these conditions, the HCT-8 cells were able to organize into tissue-like structures, including the formation of an apical brush border, tight junctions, and basal lamina. They were able to use this culturing system to study aspects of the pathophysiology of human cryptosporidiosis and demonstrate that the parasite load increased significantly in the three-dimensional intestinal model compared to standard HCT-8 cell cultures, but were limited by the short duration of time in which *C. parvum* could be maintained and could not enable the completion of the life cycle [58]. Subsequent three-dimensional intestinal models have mimicked the in vivo morphology of intestinal epithelial cells to varying degrees and have enabled the completion of the *C. parvum* life cycle in vitro. However, each of these *C. parvum* culturing systems have their limitations.

### 3.1. Perfusion Intestinal Models

**Figure 2:**
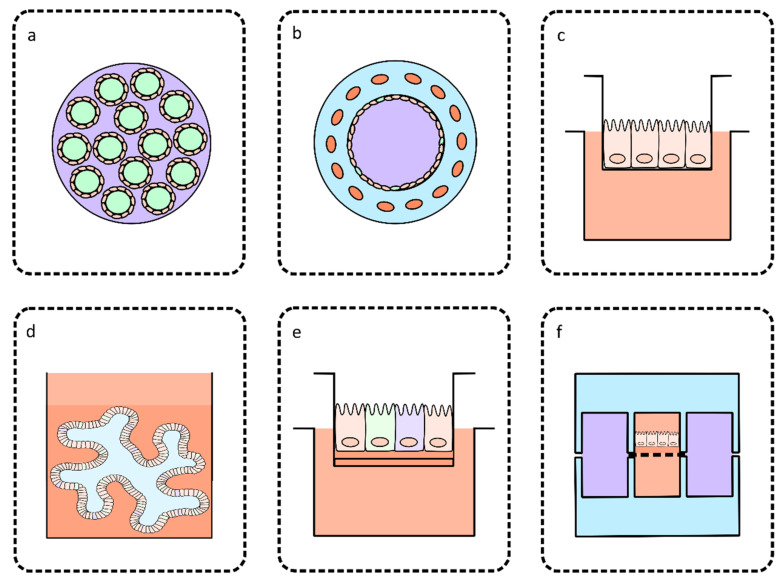
Summary diagram of the main three-dimensional intestinal models described in this review. (**a**) Cross-section of a hollow fiber cartridge described in Morada et al. [53] with hollow fibers containing host cell-specific medium in lime green, intestinal epithelial cells on the surfaces of each hollow fiber shown in peach and *C. parvum*-specific medium in the extra-capillary space shown in purple. (**b**) Cross-section of the silk protein scaffold model described in DeCicco RePass et al. [54] indicating the silk protein bulk space in blue, fibroblasts in the bulk space in orange, enterocytes and goblet cells on the luminal surface in peach and lime green respectively, and growth medium in the lumen in purple. (**c**) Mouse colon explant described in Baydoun et al. [55] maintained under air-liquid interface culturing conditions with the explant layer shown in peach and growth medium shown in orange. (**d**) Small intestine organoid described in Heo et al. [56] showing the organoid cultivated in a Matrigel layer indicated in orange and a layer of growth medium shown in peach. (**e**) Stem cell derived air-liquid interface culture described in Wilke et al. [57] showing growth medium in peach, fibroblast, and Matrigel layers in orange and each intestinal epithelial cell type in different colors. (**f**) Gut-on-a-chip described in Kim et al. [59] indicating PDMS layers in blue, vacuum chambers in purple, basal, and apical chambers divided by a semi-permeable membrane with growth medium shown in orange and a Caco-2 monolayer on the apical surface of the membrane shown in peach.

Hollow fiber technology was the first to build on the work of Alcantara Warren et al. [58] and utilize HCT-8 cells inside a three-dimensional culturing system, with the experiment taking place within a hollow cylindrical cartridge containing several hollow fibers that run through its length (Figure 2a). HCT-8 cells were cultured on the outer surface of each hollow fiber and were supplied with a growth medium optimized for host cell growth from within each hollow fiber. The host cell-specific medium was formulated to be oxygen and nutrient-rich and reached the HCT-8 cells from the basal surface. A pump system enabled the host-cell specific medium to be continuously refreshed. The compartmentalization of host cell-specific medium opened the opportunity for a separate growth medium optimized for *C. parvum* growth to occupy the extra-capillary space. The *C. parvum*-specific growth medium was developed to mimic the conditions of the human intestinal lumen, in terms of nutrient and oxygen concentration and redox conditions. The separate control of the medium supplied to both host cells and *C. parvum* was hypothesized to be key to the success of this model, which reported production of approximately 10^8^ oocysts per mL per day and continuous culture of *C. parvum* for over six months. The oocysts produced in vitro were infectious to two different types of immunodeficient laboratory mice, with oocyst shedding at a volume similar to infection initiated by commercially acquired oocysts. This system was an exciting advancement in *Cryptosporidium* biology, providing a source of fully infectious oocysts for downstream in vitro and in vivo studies [53].

Limitations of the hollow fiber technology for the culture of *C. parvum* include the requirement for expensive and specialized equipment. This approach relies on transformed cell lines rather than primary cells and despite the practical advantages of using transformed cell lines—including lower cost, higher reproducibility, and ease of use—it has been argued that transformed cell lines cannot completely capture the host-parasite interactions that occur in vivo [55,56,57]. Additionally, the cartridge needs to be disassembled to access the cells growing on the hollow fiber surface for the application of basic imaging techniques, meaning that it cannot be used to study individual stages of the *C. parvum* life cycle or host-parasite interactions over time [53]. Furthermore, high-volume production of infectious oocysts in vitro using this system has yet to be replicated by other laboratories.

### 3.2. Models Based on Co-Culture

A smaller-scale three-dimensional intestinal model was bioengineered using silk protein scaffolds that supported the co-culture of human epithelial colorectal adenocarcinoma (Caco-2) cells and mucous-secreting goblet cells (HT29-MTX). This intestinal model was fabricated using a viscous silk protein solution poured into a cylindrical polydimethylsiloxane (PDMS) mold and fashioned into a porous bulk space with a hollow lumen (Figure 2b). Caco-2 cells and HT29-MTX cells were cultured onto the luminal surface, and primary human intestinal myofibroblasts were injected into the bulk space. The myofibroblasts from the bulk space secreted cytokines and growth factors that promoted the expansion and differentiation of the cells cultured on the luminal surface. As a result, the Caco-2 cells differentiated into enterocyte-like cells containing brush borders and tight junctions and performed some of the functions observed in vivo, such as the production of alkaline phosphatase and sucrose isomaltase secretion. *C. parvum* infection was supported in this model for 15 days, during which time the parasite load was stable. Some aspects of the pathophysiology of cryptosporidiosis, such as distortion and loss of microvilli, were also observed. The luminal contents of the intestinal model were also able to be passaged three times to fresh intestinal models and re-establish infection [54]. This culture system was only able to produce small numbers of oocysts in vitro, and did not demonstrate that the oocysts produced in vitro can establish infection in vivo, which the hollow fiber technology was able to achieve [53]. However, this model does show that the co-culture of Caco-2 cells, HT29-MTX cells and primary human intestinal myofibroblasts can reproduce many aspects of the in vivo structure and function of human intestinal epithelial cells, and can serve as a model to study at least some aspects of the *C. parvum* multiplication, host-parasite interactions and the pathophysiology of cryptosporidiosis.

### 3.3. Models Based on Primary or Stem Cell-Derived Cultures

A recent study utilized colon explants from adult mice with severe combined immunodeficiency disease in a *C. parvum* culturing system (Figure 2c), building on the works of Castellanos-Gonzalez et al. [60] who were the first to use primary intestinal epithelial cells for this purpose. The three-dimensional structure of the intestinal epithelial layer of the colon explants were able to be preserved for 35 days, including the presence of microvilli, villi-like, and crypt-like structures, connective tissue with collagen, fibroblasts, and smooth muscle cells. This model was able to support *C. parvum* infection for 27 days if the initial inoculum was very low (25 oocysts per explant) [55]. While this model may not be useful for the production of large amounts of infectious oocysts, its ability to support infection of a very low number of oocysts makes it an accurate representation of what occurs in vivo during *C. parvum* infection, which is often initiated by a very small inoculum. This quality also indicates that this technology may be a promising option to be adapted as a highly sensitive detection assay for *C. parvum*. The use of primary cells in this culturing system allowed for more relevant analysis of host-parasite interactions, including the identification of lesions indicative of intraepithelial neoplasia as a result of *Cryptosporidium* infection, which had not been explored in *C. parvum* culturing systems described prior to that study.

Organoids are stem cell-derived, three-dimensional clusters of cells that resemble the in vivo structure and function of organs. Human intestinal organoids can originate from human biopsies or induced pluripotent stem cells and can faithfully reproduce the structure of intestinal epithelium [61,62]. The first study to utilize an organoid-based culturing system for the study of *C. parvum* biology used human biopsy-derived small intestine and lung organoids, which were able to support *C. parvum* growth throughout both the asexual and sexual stages of its life cycle (Figure 2d). Additionally, oocysts could be passaged within organoids for up to 28 days. *C. parvum* infection was most efficient in small intestine organoids in comparison to the lung organoids, and mainly infected differentiated enterocytes. New oocysts were produced in vitro, and these were able to produce in vivo infection in neonatal mice. The type I interferon pathway was upregulated in both lung and small intestine organoids 72 h post-infection with *C. parvum*, mirroring the host defenses observed in human cryptosporidiosis. This model was unable to produce large numbers of oocysts but was the first study to successfully utilize stem cell-derived organoids for the study of *C. parvum* and provided the only available model to study the pathophysiology of both respiratory and gastrointestinal cryptosporidiosis in humans [56].

A recent study using a mouse ileum stem cell-derived culture propagated under air-liquid interface conditions supported *C. parvum* growth throughout its entire life cycle for over 20 days while also producing between 100 and 1000 oocysts per day [57]. Air-liquid interface conditions allowed for the supply of medium to the culture from the basal surface, with the apical surface of the cells exposed to air (Figure 2e). This study utilized a complex experimental design, including the establishment of a mouse fibroblast cell monolayer on a Matrigel coated porous membrane, on top of which the stem cells were cultured. After establishing air-liquid interface culturing conditions, the stem cells differentiated into enterocytes with brush borders and tight junctions and mucous secreting goblet cells over the following two weeks. Following *C. parvum* infection of the differentiated monolayer, parasite load (as measured by qPCR) increased for 20 days to a 100-fold increase from the initial inoculum. Transcriptomic analysis of the monolayer grown under air-liquid interface conditions compared to non-air-liquid interface conditions revealed that a number of cell cycle regulation and metabolic pathways were upregulated in cells grown under air-liquid interface conditions [57]. This study also found that secretory cell expression was not required for robust *C. parvum* growth in vitro after a cell monolayer derived from an *Atoh1* knockout mouse (therefore not expressing transcription factor Atoh1 required for intestinal epithelial cells to differentiate into Paneth, goblet, and enteroendocrine cells) also supported *C. parvum* infection [57,63]. Oocysts produced in vitro were infectious and able to establish *C. parvum* infection in highly susceptible IFN-γ receptor deficient mice and re-establish infection in a fresh air-liquid interface monolayer.

The main factor associated with the large output of infectious oocysts in an in vitro culture system appears to be centered on the adequate supply of oxygen and nutrients to the host cells, whether these are primary intestinal epithelial cells or a transformed cell line. Both Morada et al. [53] and Wilke et al. [57] supplied medium to host cells from the basal surface upwards, and utilized either an oxygen-rich medium or an experimental set-up that allowed host cells to be exposed to air. Wilke et al. [57] argued that shifting host cell metabolism to favor oxidative phosphorylation over glycolysis may possibly have an important role in supporting robust growth of *C. parvum.* Other factors such as a low oxygen luminal environment to promote *C. parvum* growth, the presence of differentiated intestinal secretory cells and the use of primary intestinal epithelial cells over transformed cells appeared less important when the goal was to produce large quantities of functionally relevant *C. parvum* oocysts.

The maximum amount of time that any of the described culturing systems have been able to support continuous *C. parvum* growth is 40–45 days with passaging, with the exception of hollow fiber technology [54,57]. If using a three-dimensional model to study host-parasite interactions and the pathophysiology of cryptosporidiosis, the presence of differentiated enterocytes and the ability to apply imaging techniques to the culturing system is of the utmost importance. All of the three-dimensional culturing systems were able to reproduce the structural characteristics of enterocytes such as tight junctions and brush borders, but most either lack the ability to produce large numbers of infectious oocysts or visualize host-parasite interactions over time. A summary of the current in vitro culturing methods for *Cryptosporidium* is shown in Table 1.

## 4. Organ-on-a-Chip Technology

While most groups currently working towards continuous culture of *Cryptosporidium* are using bioengineered intestinal models, there are currently no published reports utilizing microfluidic organ-on-a-chip technology for this purpose. Organ-on-a-chip technology aims to reproduce the key features of the three-dimensional structure and function of specific tissues in the body on a miniaturized scale in vitro. The use of microfluidics enables fine control of the cellular microenvironment, including control of fluid flow through the device, shear stress on the cells, and input of mechanical stimuli [64]. Mimicking the mechanical cues that cells experience in vivo to cells cultured in vitro has been demonstrated to promote cell differentiation and formation of the three-dimensional architecture of in vivo tissues [65]. A microfluidic organ-on-a-chip device aiming to mimic the human intestine may be able to provide the ideal conditions to enable *Cryptosporidium* growth in vitro and is small-scale and affordable enough to be used in a variety of applications in the study of *Cryptosporidium* biology.

The first in vitro microfluidic devices aiming to reproduce the three-dimensional structure of the intestinal epithelium were designed for pharmaceutical testing. Common features integrated into the design of the devices included two-compartment culture chambers fabricated from PDMS, with the basolateral and apical chambers divided by a flat, semi-permeable membrane. These microfluidic devices accommodated either Caco-2 cell monolayer formation or Caco-2/HT29-MTX co-cultures, the latter of which enabled mucous layer development. Advantages of using microfluidic devices over standard cell culture included the ability to establish more physiologically relevant culturing conditions, such as the application of fluid flow and shear stress on cells, which in turn increased the capacity for long term maintenance of the cell monolayer. More practical advantages included the lower cell and reagent requirements, sparing of laboratory animals and the potential for high throughput experiments [66,67,68]. While the potential for these microfluidic devices to overcome some of the obstacles associated with standard cell culture was evident, these earlier studies often struggled with reproducibility of results, indicating that further refinement of the device design was necessary.

A subsequent study constructed a human gut-on-a-chip microfluidic device that enabled Caco-2 cells to differentiate into all four of the major intestinal epithelial cell lineages (enterocyte, mucous-secretory, enteroendocrine, and Paneth cells) and spontaneously form villi-like structures. The overall design of the microfluidic device was similar to its predecessors but incorporated additional lateral vacuum chambers that could induce peristalsis-like contractions of the semi-permeable membrane (Figure 2f). The apical surface of the membrane was coated with an extracellular matrix solution containing rat collagen and Matrigel before Caco-2 cells were cultured on top. Within this gut-on-a-chip environment, Caco-2 cells differentiated after three days of culture compared to 21 days of static culture. The cells also adopted a columnar shape. Scanning electron microscopy showed that the villi-like structures were lined with cells containing apical microvilli forming brush borders with tight junctions between each cell. The majority of the villi were composed of enterocytes, with a small number of goblet cells and Paneth cells, which was consistent with the intestinal epithelial cell population observed in vivo. The crypt-villus units observed in this gut-on-a-chip microfluidic model were able to mimic the in vivo situation where rapidly proliferating cells are found in the crypt regions and differentiate as they migrate upwards towards the villus tip [59,65]. The spontaneous formation of villi-like structures removed the requirement for custom fabricated porous membranes that reproduced the surface structure of villi seen in a previous microfluidic device [69]. As expected, under static culturing conditions, Caco-2 cells grew flat and had a squamous shape.

The unique features displayed by the Caco-2 cell monolayers cultured within this microfluidic device were mainly attributed to a combination of mimicking intraluminal fluid flow and peristalsis-like motions. Interestingly, removing peristalsis-like contractions from the gut-on-a-chip did not result in significant loss of the columnar cell shape, which indicates that the fluid flow and resulting application of shear stress on the cells is the key factor for the maintenance of physiological cell morphology [59,65]. The key role of the microfluidic shear stress on Caco-2 cell monolayers was recently illustrated and systematically investigated, stressing the need to finely control the microfluidic culture conditions [70].

Based on the findings from that study, it was established that the local mechanical microenvironment drive the differentiation of Caco-2 cells, producing a highly physiologically relevant intestinal model that even demonstrated the ability to accommodate commensal bacterial infection [65]. While it is clear that human gut-on-a-chip models can accurately reproduce the in vivo structure and function of the human intestinal epithelium, the question is raised as to whether this technology is able to support the growth of an enteric pathogen that is difficult to cultivate under standard approaches. A recent study successfully used the microfluidic device described by Kim et al. [59] to support the growth of *Shigella flexneri* and enhance bacterial invasion by 10,000-fold when compared to a Caco-2 cell monolayer cultured statically. The results of this study indicate that the combination of peristaltic motions, application of physiological fluid flow rates and, most importantly, the three-dimensional organization of Caco-2 cells into villus- and crypt-like structures, were mandatory for *S. flexneri* to efficiently infect the Caco-2 cell monolayer. Additionally, this gut-on-a-chip model was highly sensitive to *S. flexneri* infection with a bacterial load of 100 being sufficient to produce robust infection [71]. The results of this study provide a promising methodological justification for using organ-on-a-chip technology to overcome the current obstacles in culturing *Cryptosporidium*.

While most studies have utilized the Caco-2 cell line in organ-on-a-chip devices aiming to mimic the in vivo intestine, several studies used primary intestinal epithelial cells. The first primary cell microfluidic culture study used intestinal tissue slices from male Wistar rats. The slices were grown inside a microfluidic device that was designed to enable the tissue slice to be suspended in growth medium. Other features of this device included the control of fluid flow rate with syringe pumps, polycarbonate filters at the entrance and exit of the tissue chamber to ensure even distribution of medium and PDMS membranes forming gas permeable barriers allowing oxygen and carbon dioxide from the environment to enter the growth medium. Despite the controlled fluid flow enabling the growth medium to be continuously refreshed without damaging the intestinal tissue, morphological characteristics of healthy intestinal epithelial tissue such as the presence of villi were lost after only three hours of culture [72]. A subsequent study utilized primary intestinal epithelial cells in a multiorgan-on-a-chip device through the use of cells from the ileum of a human donor. The device described in that study was designed for pharmaceutical testing and contained endothelial cells cultured on the microchannel walls to mimic the human vasculature, peristaltic pumps integrated into the chip to control fluid flow, and a human liver mimic in addition to the intestinal model. Differentiation was induced over 14 days outside the microfluidic device using air-liquid interface culturing conditions, following which the primary cells were then viable within the microfluidic device for an additional 14 days [73].

More recently, studies have been integrating human intestinal organoids into organ-on-a-chip devices to mimic the in vivo human intestine. In one study, induced pluripotent stem cells were differentiated into human intestinal organoids and incorporated into a microfluidic device comprising a dual chamber system separated by a Matrigel coated semi-permeable membrane. The organoid formed a confluent monolayer after 72 h. It was found that the incorporation of fluid flow into the microfluidic device enhanced the three-dimensional structure of the organoid when compared to static culturing conditions, and after 14 days, villi formation was observed. All four major intestinal epithelial lineages were present. A gene expression study showed that when incubated with IFN-γ, a number of genes associated with inflammatory bowel disease were upregulated, which was not the case in the same device containing Caco-2 cells. This provided evidence that a human intestinal organoid-based model can more faithfully reproduce in vivo responses compared to a Caco-2-based model [74].

Another study used human biopsy-derived intestinal organoids in the context of an organ-on-a-chip microfluidic device to mimic the human small intestine. This experimental set-up used the standard PDMS-based dual chamber approach separated by an extracellular matrix-coated semi-permeable membrane and additionally cultured endothelial cells on the luminal surfaces of the basolateral chamber to imitate human vasculature [75]. The lateral vacuum chambers and syringe pumps described in Kim et al. [59] were incorporated to initiate peristalsis-like contractions of the semi-permeable membrane and fluid flow respectively. Organoids derived from the duodenal region of the small intestine were expanded and cultured in the apical chamber, where they formed villi-like structures over 8–12 days. Confocal microscopy confirmed brush border and tight junction formation. A transcriptomic approach was used to compare gene expression patterns between the gut-on-a-chip, organoid cultured under static conditions and the organoid-based intestine-on-a-chip described by Kasendra et al. [75] and showed that the transcriptome from the organoid-based intestine-on-a-chip was more similar to a human in vivo intestinal transcriptome than the intestinal organoid or the Caco-2 cell based gut-on-a-chip.

A combination of organ-on-a-chip and organoid technology as described above may be able to overcome the current obstacles in continuous culture of *Cryptosporidium*. Additionally, these systems are small scale, inexpensive, and reproducible enough to be used in a variety of applications (Table 1). Utilizing stem cell-derived cultures will build upon the works of Heo et al. [56] and Wilke et al. [57] who were able to use this approach to further the knowledge of *Cryptosporidium* biology and study the pathophysiology of cryptosporidiosis, though are still limited by the practical difficulties in recovering oocysts for reinfection in the case of organoids and relatively low parasite amplification in both studies [56,57]. The integration of organ-on-a-chip technology will make this approach more accessible to both researchers and industry and to facilitate high-throughput experiments which will aid detection assays, potentially for public health monitoring, and infectious assays, that can be used for screening for potential drug targets.

## 5. Conclusions and Future Directions

The recent successes in the long-term culturing of *C. parvum* using three-dimensional intestinal models and both mouse and human-derived intestinal organoids have laid the foundation for future expansions in the study of *Cryptosporidium* biology and elucidation of host-parasite interactions, that at present are not well understood. Further advances in these contemporary culturing techniques based on organ-on-a-chip technology may enable high-throughput discovery of drug targets in addition to forming the basis for detection and viability assays that are paramount to outbreak prevention. The lack of the ability to easily culture both *C. hominis* and *C. parvum* has been a major barrier in advancing the current knowledge of *Cryptosporidium* biology and overcoming current obstacles in reducing the global burden of cryptosporidiosis. By accurately reproducing the structure and function of the human intestinal epithelia, this new culturing technology may be able to address this issue for both *C. hominis* and *C. parvum*, providing a promising solution to a major barrier in *Cryptosporidium* research.

## Figures and Tables

**Table 1:** Summary table of the advantages and limitations of current in vitro culturing methods of *C. parvum*.

In Vitro Culture Method	Full Multiplication Cycle Supported	Maximum Time Growth is Supported	Uses		Reference
			Host–Pathogen Interactions	Large Scale Oocyst Production	
HCT-8 cell lines	No	25 days	Partially	No	Hijjawi et al. [35]
Cell-free culture	Yes	46 days	No	Partially	Hijjawi et al. [28]
Hollow fiber technology	Yes	<180 days	Partially	Yes	Morada et al. [53]
Silk-protein scaffold model	Yes	15 days	Partially	No	DeCicco RePass et al. [54]
Colon explants	Yes	27 days	Yes	No	Baydoun et al. [55]
Lung and small intestine organoids	Yes	28 days	Yes	No	Heo et al. [56]
Stem cell-derived cultures	Yes	<20 days	Yes	No	Wilke et al. [57]
